# Sleep Quality in Family Caregivers and Matched Non-Caregiving Controls: The REGARDS Study

**DOI:** 10.1093/geroni/igab046.2987

**Published:** 2021-12-17

**Authors:** Marcela Blinka, Adam Spira, Orla Sheehan, Tansu Cidav, J David Rhodes, Virginia Howard, David Roth

**Affiliations:** 1 Johns Hopkins University, Baltimore, Maryland, United States; 2 Johns Hopkins Bloomberg School of Public Health, Baltimore, Maryland, United States; 3 Johns Hopkins University School of Medicine, Johns Hopkins University, Maryland, United States; 4 Johns Hopkins University School of Medicine, Baltimore, Maryland, United States; 5 University of Alabama at Birmingham, University of Alabama at Birmingham, Alabama, United States; 6 University of Alabama at Birmingham, Birmingham, Alabama, United States

## Abstract

The high levels of stress experienced by family caregivers may affect their physical and psychological health, including their sleep quality. However, there are few population-based studies comparing sleep between family caregivers and carefully-matched controls. We evaluated differences in sleep and identified predictors of poorer sleep among the caregivers, in a comparison of 251 incident caregivers and carefully matched non-caregiving controls, recruited from the national REasons for Geographic and Racial Differences in Stroke (REGARDS) Study. Incident caregivers and controls were matched on up to seven demographic and health factors (age, sex, race, education level, marital status, self-rated health, and self-reported serious cardiovascular disease history). Sleep characteristics were self-reported and included total sleep time, sleep onset latency, wake after sleep onset, time in bed, and sleep efficiency. Family caregivers reported significantly longer sleep onset latency, before and after adjusting for potential confounders, compared to non-caregiving controls (ps < 0.05). Depressive symptoms in caregivers predicted longer sleep onset latency, greater wake after sleep onset, and lower sleep efficiency. Longer total sleep time in caregivers was predicted by employment status, living with the care recipient, and number of caregiver hours. Employed caregivers and caregivers who did not live with the care recipient had shorter total sleep time and spent less time in bed than non-employed caregivers. Additional research is needed to evaluate whether sleep disturbances contributes to health problems among caregivers.

